# Dairy-Related Dietary Patterns, Dietary Calcium, Body Weight and Composition: A Study of Obesity in Polish Mothers and Daughters, the MODAF Project

**DOI:** 10.3390/nu10010090

**Published:** 2018-01-16

**Authors:** Lidia Wadolowska, Natalia Ulewicz, Kamila Sobas, Justyna W. Wuenstel, Malgorzata A. Slowinska, Ewa Niedzwiedzka, Magdalena Czlapka-Matyasik

**Affiliations:** 1 Department of Human Nutrition, Faculty of Food Sciences, University of Warmia and Mazury in Olsztyn, 10-718 Olsztyn, Poland; lidia.wadolowska@uwm.edu.pl (L.W.); kamila.sobas@ujk.edu.pl (K.S.); justyna.wuenstel@uwm.edu.pl (J.W.W.); malgorzata.slowinska@uwm.edu.pl (M.A.S.); ewa.niedzwiedzka@uwm.edu.pl (E.N.); 2 Institute of Public Health, The Faculty of Medicine and Health Sciences, The Jan Kochanowski University in Kielce, 25-317 Kielce, Poland; 3 Institute of Human Nutrition and Dietetics, Faculty of Food Science and Nutrition, Poznan University of Life Sciences, 60-624 Poznan, Poland; magdalena.matyasik@up.poznan.pl

**Keywords:** body fat, BMI, calcium, dairy products, dietary patterns, obesity, waist circumference, WHtR

## Abstract

The role of the family environment in regards to dairy products and dietary calcium in the context of obesity is not fully understood. The aim of the study was to investigate the association among dairy-related dietary patterns (DDPs), dietary calcium, body weight and composition in mothers and daughters. Data were collected through a cross-sectional survey within the MODAF Project. A total sample of 712 pairs of mothers (<60 years) and daughters (12–21 years) was studied. This study included 691 pairs. A semi-quantitative food frequency questionnaire (ADOS-Ca) was used to collect dietary data. Waist circumference (WC), body fat, waist-to-height ratio (WHtR) and body mass index (BMI) were determined. Previously derived DDPs were used—three in mothers and three in daughters. In mothers, two of the DDPs were characterized by higher consumption of various dairy products with suboptimal calcium content (means: 703 or 796 mg/day) which decreased the chance of: *z*-WC > 1 standard deviation (SD), WC > 80 cm, body fat > 32%, WHtR > 0.5, BMI = 25–29.9 kg/m^2^ or BMI ≥ 30 kg/m^2^ by 44–67% when compared to low-dairy low-calcium DDP (288 mg/day). In mothers per 100 mg/day of dietary calcium, the chance of *z*-WC > 1SD, WC > 80 cm, *z*-WHtR > 1SD, WHtR > 0.5 cm, BMI = 25 to 29.9 kg/m^2^ or BMI ≥ 30 kg/m^2^ decreased by 5–9%. In correspondence analysis, a clear association was found between mothers’ and daughters’ low-dairy low-calcium DDPs and upper categories of *z*-WC (>1 SDs). This study reinforces evidence of the similarity between mothers and daughters in dairy-related dietary patterns and provides a new insight on the adverse relation between low-dairy low-calcium dietary patterns and obesity. It was found that diets containing various dairy products with suboptimal dietary calcium content may be recommended in obesity prevention.

## 1. Introduction

Dietary calcium and dairy products have been extensively studied in the context of various diet-related diseases. It has been shown that the consumption of dairy products helps to meet dietary recommendations and may protect against the most prevalent chronic diseases, whereas very few adverse effects have been reported [1,2]. Low consumption of dairy products was associated with increased risk of heart disease [1,2] dyslipidemia [3,4] hypertension [1,4,5,6] metabolic syndrome [1,2,7,8] low bone mineral density and osteoporosis [4,9]. To the contrary, high consumption of dairy products was shown to reduce the risk of metabolic syndrome by 14% and 17% in cohort and cross-sectional studies, respectively [8], diabetes type 2 [1,3] and some types of cancers such as colorectal, bladder, gastric and breast cancer [2,10].

Dairy product consumption was found to play an important role in maintaining normal body weight [11,12,13,14,15]. Since the rates of overweight and obesity have risen dramatically in the past few decades and are currently a serious public health challenge [16], the impact of the whole dairy food matrix and dietary calcium alone on body weight has gained much interest. Two main mechanisms are discussed: the effect of calcium on fatty acid absorption from the gastrointestinal tract and connecting fatty acids with intracellular calcium in adipocytes [17,18]. A possible mechanism for reducing fat deposition is improving insulin sensitivity as an effect of the suppression of calcitriol levels [13,17]. A review by Bendtsen [13] indicates that a high proportion of calories from dairy products and high calcium intake prevents body weight gain. Further studies in women, children and adolescents have reinforced these findings [19,20,21]. In contrast, Lee et al. [14] found that the consumption of at least one serving of dairy products a day (milk, yoghurt, cheese and dairy based desserts) lowered the risk of obesity in Korean, but not European, women. Further research is needed to investigate the associations between dairy product consumption and obesity risk in Europeans.

To date, most studies on dairy products and health outcomes have focused on single dairy products, such as milk, yoghurt, cheese, low-, regular- or high-fat dairy [4,7,22]. This simplistic approach, however, does not allow for a straightforward interpretation. Studying dietary patterns (DPs) appears to be a more fitting approach in finding associations between diet and diet-related diseases, since DPs better reflect the whole diet than its single characteristics. To date, dairy products have been investigated as a part of dietary patterns, which included a variety of different products, including those from other food groups [23,24]. Data regarding dietary patterns solely related to dairy products are scarce. Moreover, fewer studies were conducted on Europeans and many of them were related to Asian, South American or African countries with a traditionally low consumption of dairy products [7,14]. In Europeans, a lower obesity risk was associated with DPs which consisted of plenty of plant foods, fish, seafood and low-fat dairy products [23]. A relatively lower body mass index (BMI) was found in Hispanic adults who adhered to the Whole milk pattern when compared to participants adhering to other DPs [25]. The inverse association with BMI and waist circumference (WC) and a DP high in reduced-fat dairy products (healthy DP) was found in a longitudinal study among adult Caucasian Americans [26]. Thus, no clear conclusion has been drawn in regards to obesity risk and dietary patterns focused on dairy products [24].

The environment is one of the important factors that affect people’s dietary behaviors, reflected in dietary patterns. In particular, it has been shown that parental eating behaviors can have a strong impact on what is consumed by their children [27]. It was previously documented that mothers can influence their daughters’ dietary behaviors either positively (implementing healthy habits) or negatively (developing dietary restrictions and eating disorders) [28,29]. Current findings regarding the associations among the environment, dietary patterns and obesity are inconclusive. A randomized controlled trial showed that physical and social environment influenced DPs but did not prevent overweight or obesity [30]. In South African mothers and daughters with similar knowledge, diet perceptions and physical activity, no similarities were found in terms of BMI [31]. Furthermore, a relationship was found between eating healthier family meals and lower BMI [32,33]. Maternal BMI was a modest predictor of daughters’ relative body weight [28,34]. Some studies were focused on family environment and body weight status or obesity but without dietary context [34]. Pachucki et al. [35] found that the obesity status of different children within the same family is related to a parent or sibling’s obesity.

All findings demonstrated family similarities in the context of obesity with the reference to foods or eating behaviors, however studies referring to dietary patterns related to dairy products are missing. To fill this gap, it is worth studying the association between obesity risk and dairy-related dietary patterns (DDPs) previously found in mothers and daughters [33]. The aim of the current study was to investigate the association between DDPs, dietary calcium and body weight and composition in mothers and daughters. It was hypothesized that the similarity within families previously found in mothers and daughters in terms of consumption of dairy products and dietary calcium also exist in terms of body weight and composition as a result of similar DPs.

## 2. Materials and Methods

### 2.1. Study Design and Participants

Analyses were carried out on data from the project: “Analysis of mother—daughter dairy products food patterns in relation to bone mineral status and calcium deficiency and osteoporosis risk among women. MODAF Study”. It was cross-sectional study design and the data was collected by well-trained researchers in 2007–2010.

**Figure 1:**
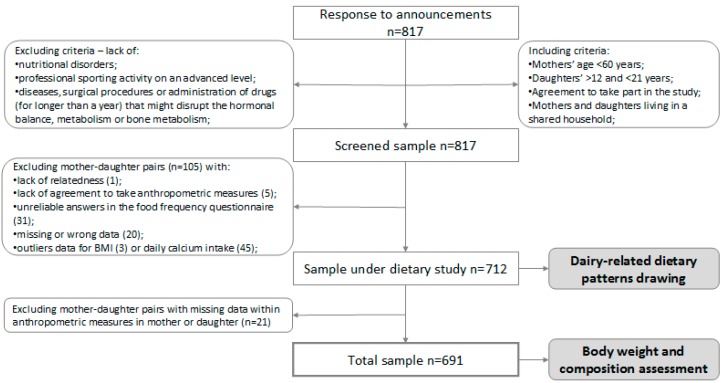
Flowchart: Study design and data collection.

Mothers (<60 years) and their daughters (12–21 years) living in a shared household were recruited within the framework of the MODAF study. Details regarding the recruitment protocol have been previously reported [36,37]. In brief, the participants were a convenience sample. The including and excluding criteria are presented in Figure 1. From the screened sample (*n* = 817), 105 mother—daughter pairs were excluded, mainly due to missing data, unreliable answers in the food frequency questionnaire and outliers data. The sample under dietary study included 712 mother—daughter pairs [36]. After exclusion of a further 21 mother—daughter pairs with missing anthropometric measurement data, the final sample size was 691 pairs.

### 2.2. Dietary Assessment and Dairy-Related Dietary Patterns Drawing

To collect dietary data, a semi-quantitative food frequency method was applied. A validated a semi-quantitative food frequency questionnaire (ADOS-Ca) was used to evaluate the consumption of dairy products, calcium intake from dairy products and daily intake [38]. Details regarding dietary data collection have been previously reported [37]. In brief, respondents indicated the frequency of consumption and portion size of 11 items of dairy products habitually eaten during the last six months. Dietary calcium from the daily diet (mg/day) was estimated by: (i) summing up the calcium intakes from 11 items of dairy products; AND (ii) using the formula worked out in the validation study to convert calcium intake from dairy products to calcium intake from daily diet [38]. To assess dietary calcium intake, the cut-off point method was used [39]. The number of mothers and daughters (in %) who did not meet the Polish calcium intake recommendations, i.e., with calcium intake below Estimated Average Requirement (EAR), was calculated. For Polish females, the EAR of calcium intake is 1100 (10–18 years), 800 (19–50 years) and 1000 mg/day (>51 years) [39].

DDPs were separately derived for mothers and daughters. Principal Component Analysis (PCA) and cluster analysis (k-means method) were applied. Details regarding DDPs have been previously reported [36]. In brief, the input variables in the PCA were calcium intakes (mg/day) from 11 items of dairy products, and in the cluster analysis—the factors extracted in the PCA (five factors in mothers, six factors in daughters). Three clusters were found in both mother and daughter samples. These clusters identified DDPs.

In mothers were found: “Common”, “Cheese and fruit yoghurt” and “Natural milk beverages and cottage cheese” patterns; and, in daughters: “Common”, “Yoghurt and cheese” and “Milk and cheese” patterns (Table 1). In mothers, the “Common” pattern (69% of mothers) was characterized by low a consumption of all dairy products and calcium (mean calcium intake 288 mg/day; 100% of mothers below EAR); “Cheese and fruit yoghurt” pattern (13% of mothers) was characterized by the highest calcium intake from rennet cheese, fruit yoghurt, ice-creams and cream (703 mg/day; 77% of mothers below EAR); and “Natural milk beverages and cottage cheese” pattern (18% of mothers) was characterized by the highest calcium intake from milk, natural yoghurt, kefir/buttermilk, cottage cheese, homogenized cheese, processed cheese and cheese for spreading (796 mg/day; 59% of mothers below EAR). In daughters, “Common” pattern (67% of daughters) was characterized by low consumption of all dairy products and calcium (373 mg/day; 97% of daughters below EAR); “Yoghurt and cheese” pattern (14% of daughters) was characterized by the highest calcium intake from fruit yoghurt, natural yoghurt, kefir/buttermilk, homogenized cheese and high calcium intake from rennet cheese (808 mg/day; 72% of daughters below EAR); and “Milk and cheese” pattern (19% of daughters) was characterized by the highest calcium intake from milk, processed cheese, cream and high calcium intake from rennet cheese (901 mg/day; 63% of daughters below EAR).

### 2.3. Body Weight and Composition Assessment

Measurements of body weight (kg), height (cm), WC (cm) and body fat (%) were taken using professional devices and measuring tape. Body fat was measured by FUTREX 6100 (F6100/XL, Futrex; Hagerstown, MD, USA). All measurements were taken in light clothing and without shoes and WHtR and BMI (kg/m^2^) were then calculated. Central obesity was identified for WHtR ≥ 0.5 in accordance with Ashwell et al. [40] or WC > 80 cm in accordance with Alberti et al. [41]. The same cut-off of WC was used for all females, because there is a lack of specific criteria for females <18 years. For this reason, it was decided that this cut-off could be used temporarily. BMI was categorized in accordance with the International Obesity Task Force (IOTF) standards for adult females and according to age-sex-specific BMI cut-offs for females <18 years [42]. Finally, BMI of all females was classified as follows: underweight (<18.5 kg/m^2^), regular weight (18.5 to 24.9 kg/m^2^), overweight (25 to 29.9 kg/m^2^) and obesity (≥30 kg/m^2^). Body fat for adult females was categorized in accordance with Taton recommendations [43] as follows: low body fat (<14%), regular body fat (14–28%), overweight (29–32%), and obesity (>32%). *Z*-scores (in SDs) of WC, body fat, WHtR and BMI were calculated using the authors’ own database, separately for mothers and daughters. *Z*-scores (*z*-WC, *z*-Body fat, *z*-WHtR, *z*-BMI) were categorized as follows: bottom category (<−1 SD), central category (−1 to 1 SD), and upper category (>1 SD).

### 2.4. Confounding Factors

Socioeconomic variables and factors related to dietary habits, bone health and body composition were collected. More details have been previously reported [36,44]. In brief, the standard questions from the ADOS-Ca questionnaire were applied. Confounding factors were characterized as follows: education (elementary, secondary, and high), place of living (rural and urban), economic situation (bad, satisfactory, good, and very good), chronic disease (yes or no), menstrual cycle (yes or no), daily consumption of dairy products during pre-school period (yes or no), daily consumption of dairy products during school period (yes or no), dieting (yes or no), taking calcium supplement (yes or no), and physical activity. To assess physical activity level, a validated International Physical Activity Questionnaire (IPAQ), long version, in the Polish language was applied [45]. Weekly activities in four domains (with 12 items) were collected: leisure time, domestic and gardening, school- or work-related and transport-related (duration in minutes and frequency in days). The physical activity level was expressed as a standard Metabolic Energy Task (MET) in MET-minutes/week [45]. The physical activity level was converted into four categories (low: <600, moderate: 600–1499, moderate-higher: 1500–2999, and high: ≥3000 MET-minutes/week).

### 2.5. Ethical Approval

The study protocol was registered and approved by: (i) the Bioethics Committee of the Regional Medical Chamber in Olsztyn in 2001, on 27 June 2001, Resolution No. 49/2001; and (ii) the Bioethics Committee of the Faculty of Medical Sciences, University of Warmia and Mazury in Olsztyn on 17 June 2010, Resolution No. 20/2010). All participants provided informed consent.

### 2.6. Statistical Analysis

Continuous variables were presented as means with 95% confidence interval (95% CI), and categorical variables as a sample percentage (%). The differences between groups were verified by chi^2^ test (categorical variables), Kruskal–Wallis test (continuous variables of dietary data) or two-tailed *t*-test (continuous variables of body weight and composition data). Variable normality was checked by a Kolmogorov–Smirnov test before analysis.

The associations between DDPs and body weight and composition were verified by a logistic regression analysis. The analysis was carried out separately for mothers and daughters. The odds ratios (ORs) and 95% CI were calculated. ORs represented the chance of the prevalence of abnormal body weight and composition associated with females’ DDPs or per 100 mg/day of dietary calcium. The reference category were mothers or daughters from “Common” patterns (OR = 1.00) and normal or central category of body weight and composition characteristics (OR = 1.00). The significance of ORs was assessed by Wald’s statistics. Two models were created: crude–without adjustment for confounders and adjusted for age, education, place of living, economic situation, chronic disease, menstrual cycle, daily consumption of dairy products during pre-school period, daily consumption of dairy products during school period, dieting, taking calcium supplements and physical activity. Details referring to confounders categories were described in Section 2.4. Missing data referring to physical activity in 292 mothers, because of unreliable reports, was supplemented by moda category and used in logistic regression.

The association between mothers and daughters referring to DDPs, body weight and composition in a complex system was verified with a multiple correspondence analysis. Four analyses were carried out separately for each *z*-characteristic (*z*-WC, *z*-Body fat, *z*-WHtR, *z*-BMI). Each analysis included 12 categories: 3 mothers’ DDPs, 3 daughters’ DDPs, 3 categories of mothers’ *z*-characteristic, 3 categories of daughters’ *z*-characteristic. The results were presented on plots in the system of two co-ordinates. All models explained 30% or 31% of inertia. On each plot, the clusters were distinguished and marked as a blue rectangle. These clusters contain points referring to categorized variables and present the association between mothers’ and daughters’ DDPs and mothers’ and daughters’ *z*-characteristic categories. The closer the points are, the greater is the association between variables.

The association between mothers’ and daughters’ body weight and composition was estimated with linear correlation analysis. Pearson correlation coefficients were calculated for *z*-characteristics of mothers and *z*-characteristics of daughters, separately for *z*-WC, *z*-Body fat, *z*-WHtR, *z*-BMI. For all tests, *p*-value < 0.05 was considered as significant. The statistical analysis was performed with Statistica 13.1 PL software (*Statistica*, version 13.1 PL; StatSoft Inc.: Tulsa, USA).

## 3. Results

### 3.1. Description of Participants’ Body Weight and Composition by DDPs

Table 2 shows the distributions of body weight and composition characteristics. In mothers, central obesity was found in 45% or 52% of females depending on the applied criterion (WHtR or WC, respectively); 24% or 38% of mothers were overweight (for body fat or BMI as criterion, respectively); and 55% or 13% of mothers were obese (for body fat or BMI as criterion, respectively). In daughters, central obesity was found in 5% or 8% of them (for WHtR or WC as criterion, respectively); 6% of daughters (for BMI as criterion) were overweight; and 1% of daughters (for BMI as criterion) were obese.

In mothers with “Cheese and fruit yoghurt” or “Natural milk beverages and cottage cheese” patterns, when compared to “Common” pattern, significantly lower means were found for: WC (by 5.1 or 4.0 cm, respectively), *z*-WC (by 0.47 or 0.37 SDs, respectively), *z*-WHtR (by 0.48 or 0.37 SDs, respectively), BMI (by 1.8 or 1.3 kg/m^2^, respectively) and *z*-BMI (by 0.43 or 0.31 SDs, respectively; Table 3). For body fat and *z*-Body fat, significant lower means were found only for mothers with “Cheese and fruit yoghurt” pattern (by 2.7% and 0.55 SDs, respectively). Significantly fewer mothers with “Cheese and fruit yoghurt” or “Natural milk beverages and cottage cheese” patterns when compared to “Common” pattern fall in the higher category of body weight and composition: *z*-WC > 1SDs (9% or 9% vs 19%, respectively), WC > 80 cm (41% or 39% vs 58%, respectively), *z*-WHtR > 1SDs (8% or 10% vs 17%, respectively), WHtR > 0.5 (32% or 35% vs 51%, respectively), and BMI ≥ 25 (41% or 40% vs 56%, respectively; Table 2).

In daughters, in respect to DDPs, no significant differences for all characteristics of body weight and composition were revealed for means as well as distributions (Table 2 and Table 3).

### 3.2. Associations among DDPs, Body Weight and Composition

Table 4 shows the adjusted ORs of the prevalence abnormal body weight and composition according to DDPs and calcium intake. In mothers, “Cheese and fruit yoghurt” pattern when compared to “Common” pattern significantly decreased the chance of central obesity (by 53% or 60%, for WC or WHtR as criterion, respectively) and obesity (by 60%) when criterion was body fat (>32%). In mothers, “Natural milk beverages and cottage cheese” pattern significantly decreased the chance of central obesity (by 71% for WC as criterion,) and overweight (by 56%) when the criterion was BMI (25–29.9). In mothers in “Natural milk beverages and cottage cheese”, the opposite association was found for bottom categories of *z*-Body fat, i.e., the chance increased by 139%.

In mothers per each 100 mg/day of dietary calcium, the chance of upper categories (>1SDs) of *z*-WC, *z*-WHtR or *z*-BMI decreased by 9–10%, central obesity by 7% (for WC as criterion), overweight by 8% and obesity by 11% (both for BMI as criterion) (Table 4).

In daughters, “Milk and cheese” pattern when compared to “Common” pattern significantly increased the chance of bottom category of *z*-WHtR (<−1 SDs) by 87% (Table 4). No association was found between daughters’ dietary calcium intake and higher ranges of characteristics of body weight and composition but some opposite associations were significant. In daughters per each 100 mg/day of dietary calcium, the chance of bottom category (<−1 SDs) increased by 6–7% for *z*-WHtR or *z*-BMI and by 5% for underweight (for BMI as criterion).

Crude ORs of the prevalence of abnormal body weight and composition according to DDPs and dietary calcium were similar to those revealed for adjusted ORs (Table S1).

The correlation between mothers’ and daughters’ *z*-characteristics of body weight and composition were weak but significant (*p* < 0.001). Pearson correlation coefficients were 0.26 (for *z*-WC, and *z*-Body fat) and 0.21 (for *z*-WHtR and *z*-BMI) (Supplementary Materials, Table S2).

**Figure 2:**
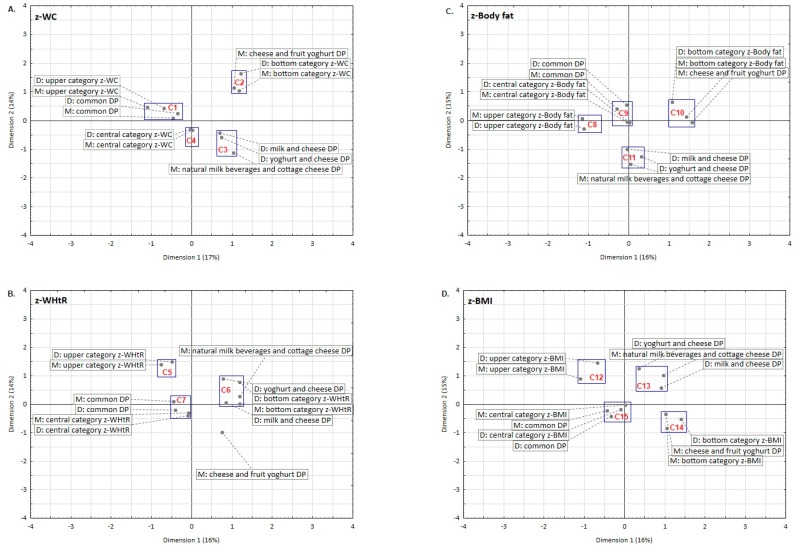
Graphical projection of a multiple correspondence analysis of the associations between body weight and composition *z*-characteristics and dairy-related dietary patterns (variance explained by each dimension). Notes: DP: Dietary pattern.

In a multiple correspondence analysis, a similarity was found between mothers and daughters in respect to DDPs and *z*-characteristics (Figure 2). Three or four clusters per each plot (C1 to C15 in total) were distinguished. *Z*-characteristics of both mothers and daughters were the most strongly associated with their “Common” patterns. Only one cluster (C1) presents an association between upper categories of *z*-characteristic (>1 SDs) and DDPs of mothers and daughters – it was revealed for upper categories of *z*-WC and “Common” patterns (Figure 2A). The points were close to each other (in the same quarter of the co-ordinate system), so the association may be interpreted as strong. Mothers’ and daughters’ “Common” patterns were also associated with central categories of *z*-WHtR (C7; Figure 2B), central categories of *z*-Body fat (C9; Figure 2C) or central categories of *z*-BMI (C15; Figure 2D). Although points within these three clusters were close to the 0 point of the two-coordinate system or lay in two quarters of the system, such an association may be interpreted as weaker. Many clusters (C2, C4–C6, C8, C10, C12, and C14) present an association between the same category of mothers’ and daughters’ *z*-characteristics and one DDPs referring to mothers or to daughters, e.g., C2: bottom categories of *z*-WC both mothers and daughters and mothers’ “Cheese and fruit yoghurt” pattern (Figure 2A); C4: central categories of *z*-WC for both mothers and daughters (Figure 2A); and C5: upper categories of *z*-WHtR both mothers and daughters (Figure 2B).

## 4. Discussion

The present study indicates that dietary calcium, as well as dietary patterns characterized by higher consumption of various dairy products with suboptimal dietary calcium levels, decreased obesity chance in mothers and increased the chance of thinness in daughters when compared with low-dairy low-calcium dietary patterns. This relation was clear in mothers but weaker in daughters. It was found that mothers and daughters with similar low-dairy low-calcium patterns and mothers and daughters with higher WC (upper categories of *z*-score) were components of the same cluster. The current evidence did not confirm the familiar similarity in terms of body weight and composition and similar dietary patterns characterized by higher consumption of various dairy products with suboptimal dietary calcium levels.

The study supports some previous findings that diets containing more dairy products and dietary calcium may prevent obesity in adult females [3,15,18]. It was found in mothers that the chance of obesity measured with various characteristics of body weight and composition lowered by 7–11% per each 100 mg of dietary calcium. The current findings are in accordance with a study carried out among Polish women aged 20–80 years [6]. Furthermore, in mothers, two dairy-related dietary patterns with suboptimal dietary calcium content (700–800 mg/day) greatly lowered the chance of central obesity (by 50–70%). One of these dietary patterns contained more calcium and consisted of low-fat dairy (“Natural milk beverages and cottage cheese”), and the second pattern contained slightly less calcium and consisted of full- and low-fat dairy (“Cheese and fruit yoghurt”). The current study is consistent with many studies and systematic reviews showing that full-fat dairy products are associated with a lower risk of obesity or central obesity [1,2,4,12] or do not increase the obesity risk [11]. Furthermore, it was concluded that current dietary recommendations to avoid full-fat dairy products are not evidence-based [1,11]. The current findings showing a health benefit of dietary pattern consisted of full- and low-fat dairy can be explained by the impact of dietary calcium alone, the whole dairy food matrix as well as dairy fat. Such a dietary pattern can prevent obesity through a variety of mechanisms, including the influence of dairy casein and lactose on increasing of calcium bioavailability, the effect of calcium and its ability to bind unconjugated bile acids and free fatty acids from the gastrointestinal tract and prevent fat absorption, reduce lipogenesis and increase lipolysis by connecting fatty acids with intracellular calcium in adipocytes [17,18]. In respect to dairy fat, its suspected health effect on obesity prevention has been attributed to conjugated linoleic acids, cis and trans palmitoleic acid, butyric acid, phytanic acid, and alpha-linolenic acid [11,12], reducing chronic inflammation and lipid peroxidation [46], and the better absorption of minerals (and calcium) from full-fat than low-fat dairy products [1,11].

In daughters, the association between obesity risk and dietary calcium or dairy-related dietary patterns was indirect and weaker than in mothers. There were fewer significant associations between the characteristics of body weight and composition and the dietary calcium and the dietary pattern. In contrast to mothers, in daughters, a higher dietary calcium intake or dietary pattern containing more calcium increased the chance of thinness. Per each 100 mg of dietary calcium, the chance of having lower ranges of some characteristics of body weight and composition increased by 5–6%. The dietary pattern (“Milk and cheese”) containing more calcium (approximately 900 mg/day) highly increased the chance (by 87%) of the lower range of WHtR, thus lowered the risk of central obesity. Indirectly, this result is well supported in the literature [2,19,21]. A cross-sectional survey in U.S. children showed that the consumption of dairy, particularly yoghurt, was associated with lower body fat [21]. Children and adolescents with the highest dairy intake were less likely (by 38%) to be overweight or obese compared to those with the lowest dairy intake [19]. Across some studies, no association between dairy intake and body weight in adolescents was found [47,48,49]. For example, Nezami et al. [49] found an association between dairy intake and body composition in boys, but not in girls. In cross-sectional study among Brazilian adolescents (11–14 years), an inverse association between DPs consisting of dairy products and overweight/obesity was found [48]. This study has also revealed that a dietary pattern which consisted of coffee and dairy products (milk, yogurt and cheese) was associated with higher risk of overweight/obesity. The current findings among daughters are partially consistent with most of the longitudinal studies reviewed by Louie et al. [20]. Out of the 10 studies reviewed, six showed no significant association, one showed an increased risk of obesity and three reported a protective association between dairy consumption and the risk of overweight or obesity [20].

A familiar similarity in terms of body weight and composition and dairy-related dietary patterns was found for low-dairy low-calcium dietary patterns only. The association was clear, but shown for only one characteristic of body composition. In the multiple correspondence analysis, one cluster grouping mothers and daughters with “Common” patterns and mothers and daughters with upper categories of WC was identified. Thus, the familiar similarity in the consumption of low-dairy low-calcium diet was associated with a higher risk of central obesity. The present study gives new insight into familiar similarity and shows a “negative face” of the family environment in terms of dairy product consumption, dietary calcium, body weight and composition. Both positive and negative influences on what their children eat for parental eating behaviors were shown [27,28,29]. The maternal impact on child eating habits was expressed by controlling what the daughter ate and pressure to eat healthier foods, developing dietary restrictions and maternal eating disorders. In the context of dairy product consumption, Fisher et al. [29] previously revealed that mothers’ milk intakes may affect their daughters’ calcium adequacy in early childhood by influencing the frequency with which their daughters consume those beverages. Because studies referring to low-dairy low-calcium dietary patterns in obesity context to find family similarities are missing, it is difficult to discuss our result.

We found a lack of familiar similarity in terms of high-dairy dietary patterns with suboptimal calcium content and body weight and composition. Thus, a “positive face” of the family environment in terms of high-dairy dietary patterns and lower obesity risk was not shown. The low accordance between mothers and daughters in the body’s response to these dietary patterns can be explained by impact of age and metabolic differences. It been shown that higher dairy intake is related to healthier dietary habits and a more balanced diet [7]. In adolescence, such diet can speed up growth and improve body composition by increasing a muscle mass with or without the lowering of fat mass [19,49]. In this study, this possible mechanism cannot be confirmed because muscle mass was not measured. In adult women, after finishing the growing period, such a diet can clearly prevent obesity, as clearly shown in the study. Further studies should be focused on a detailed assessment of body composition adolescent female and their body’s response to high-dairy dietary patterns with suboptimal calcium content.

Finally, it should be underlined that a clear familiar similarity in terms of body weight and composition was found. Since seeking this relation between mothers and daughters was not the main aim of the study, the statistical analysis was limited to a multiple correspondence analysis. Many clusters were identified which grouped mothers and daughters with the same category of characteristics of body weight and composition (bottom, central or upper). The current findings strengthen the previous results. Using BMI as overweight/obesity measure, Whitaker et al. [34] revealed that overweight or obese parents increased the risk of child obesity and the relationship was stronger between mother and child than father and child. In the current study, familiar similarity in two female generations was found for all four *z*-scores under study (WC, percentage of body fat, WHtR and BMI).

The major strengths of this study are a relatively large sample of mother and daughter family pairs (near to 1400 subjects), widely reflecting the sociodemographic status of Polish society. Secondly, various characteristics of body weight and composition were used to assess obesity risk, including international cut-offs and based on own data-set (*z*-scores). Thirdly, in statistical analysis, many confounders were applied which were potentially influencing on associations between DPs and body weight and composition characteristic, e.g., age, socioeconomic, dieting, taking calcium supplement, and physical activity. This approach reinforces the current outcomes and conclusions.

The study is not without limitations. No data were collected related to the energy value of the whole diet and fat content. These factors are two main dietary factors influencing energy balance, body mass and composition. This weakness was corrected by using an adjustment for physical activity. Despite this, the summary effect of dairy-related dietary patterns and physical activity on obesity risk could be measured. Finally, data were collected for different kinds of dairy products without diversification into low-, regular- and full-fat dairy. Although this may limit the conclusions of the study, it may simplify the recommendations drawn based on these results.

## 5. Conclusions

Based on a holistic approach by considering dietary patterns, it was found that diets containing various dairy products with suboptimal dietary calcium levels as well as dietary calcium might prevent obesity in adolescent and adult females. In this field, some specific differences between mothers and daughters were revealed. Dietary calcium and diets consisting of various dairy products with suboptimal dietary calcium levels decreased obesity chance in mothers but increased thinness chance in daughters. This relation was clear in mothers, but weaker in daughters. Future research should be focused on girls to assess new aspects of body composition, including muscle mass and biomarkers of underweight in the context of dairy-related dietary patterns and dietary calcium.

The study reinforces evidence on the similarity between mothers and daughters in dairy-related dietary patterns and gives new insight into the adverse relation between low-dairy low-calcium dietary patterns and obesity. A “negative face” of familiar similarity in terms of dairy-related dietary patterns and body weight and composition was found. The familiar similarity in consuming of low-dairy low-calcium diet was associated with a tendency to higher WC of both mothers and daughters. Summarizing, the current study provides a good basis upon which to highlight the importance of a higher consumption of various dairy products and suboptimal dietary calcium intake to prevent obesity in adolescent and adult females.

## Figures and Tables

**Table 1:** Sample characteristics by dairy-related dietary patterns (percent or mean with 95% confidence interval).

Characteristics	Mothers					Daughters				
	Total	Dairy-Related Dietary Patterns			*p*-Value	Total	Dairy-Related Dietary Patterns			*p*-Value
		Common	Cheese and Fruit Yoghurt	Natural Milk Beverages and Cottage Cheese			Common	Yoghurt and Cheese	Milk and Cheese	
	(*n* = 691)	(*n* = 477)	(*n* = 88)	(*n* = 126)		(*n* = 691)	(*n* = 463)	(*n* = 94)	(*n* = 134)	
Age (years) ^a^	44.0 (43.5; 44.4)	44.3 (43.8; 44.8)	42.2 (40.8; 43.6)	44.1 (43.1; 45.2)	ns	17.0 (16.8; 17.2)	17.1 (16.8; 17.3)	16.9 (16.4; 17.4)	16.8 (16.4; 17.3)	ns
Age groups of mothers/daughters (%)										
29–39/12–14.9 years	26	23	38	31		28	26	30	31	
40–49/15–17.9	57	60	49	52	0.041	27	27	31	25	ns
50–59/18–20.9	16	17	14	17		45	47	39	44	
Education (%)										
Elementary	30	31	25	25		41	40	39	46	
Secondary	42	43	40	41	ns	38	37	48	31	ns
High	28	26	35	33		21	23	13	23	
Place of living (%)										
Rural	51	52	57	39	0.010	51	52	57	39	0.010
Urban	49	48	43	61		49	48	43	61	
Economic situation (%)										
Bad	1	1	0	1		1	1	0	1	
Satisfactory	23	25	19	20	ns	23	25	19	20	ns
Good	67	65	70	69		67	65	70	69	
Very good	9	9	10	10		9	9	10	10	
Chronic disease (%)	17	17	22	14	ns	7	6	10	4	ns
Menstrual cycle (%)	87	87	88	83	ns	94	93	96	95	ns
Daily consumption of dairy products (%) during										
Pre-school period	64	65	63	64	ns	54	50	59	66	0.006
School period	57	57	57	59	ns	38	32	36	58	<0.001
Dieting (%)	17	16	18	21	0.032	13	13	20	8	ns
Taking calcium supplement (%)	17	16	11	19	ns	14	9	18	7	0.008
Physical activity (%) ^b,c^										
Low	32	31	30	39		16	15	16	17	
Moderate	52	53	57	43	ns	36	34	45	39	ns
Moderate-higher	15	15	13	16		18	19	12	16	
High	1	1	0	2		30	32	28	28	
Calcium (mg/day) ^a^ from:										
Milk	155 (139; 170)	105 (94; 116)	101 (79; 122)	380 (324; 435)	<0.001	161 (146; 176)	85 (77; 93)	129 (96; 162)	446 (406; 485)	<0.001
Rennet cheese	89 (79; 89)	59 (53; 64)	261 (214; 307)	82 (66; 98)	<0.001	135 (122; 147)	123 (108; 138)	153 (116; 191)	161 (130; 193)	0.619
Fruit yoghurt	58 (52; 64)	46 (41; 51)	129 (99; 159)	55 (44; 66)	0.004	101 (91; 111)	77 (68; 85)	194 (142; 246)	119 (102; 137)	0.003
Natural yoghurt	53 (45; 60)	32 (27; 38)	74 (54; 93)	115 (82; 147)	<0.001	33 (27; 38)	15 (13; 18)	135 (107; 163)	21 (14; 27)	<0.001
Kefir, buttermilk	21 (17; 24)	8 (7; 10)	19 (11; 28)	69 (55; 83)	<0.001	18 (15; 21)	9 (7; 10)	70 (51; 89)	13 (10; 16)	<0.001
Processed cheese	19 (15; 22)	11 (9; 13)	11 (9; 13)	20 (15; 24)	<0.001	29 (24; 34)	20 (16; 23)	33 (21; 45)	58 (37; 79)	0.001
Fresh cheese	15 (14; 17)	10 (9; 11)	12 (10; 15)	39 (31; 47)	<0.001	10 (8; 11)	6 (5; 17)	28 (21; 36)	39 (31; 47)	<0.001
Homogenized cheese	8 (7; 10)	6 (5; 6)	12 (9; 16)	17 (12; 21)	0.002	16 (15; 18)	9 (8; 10)	27 (20; 33)	10 (8; 13)	<0.001
Ice-creams	8 (7; 9)	7 (6; 7)	18 (13; 23)	8 (7; 10)	<0.001	28 (26; 31)	26 (24; 29)	31 (24; 39)	32 (26; 38)	0.695
Cream	5 (4; 6)	3 (3; 4)	16 (11; 21)	5 (4; 7)	<0.001	2 (2; 3)	1 (1; 1)	3 (2; 5)	5 (3; 7)	<0.001
Cheese for spreading	2 (1; 3)	1 (1; 1)	3 (1; 4)	6 (0; 12)	0.036	2 (1; 2)	2 (1; 2)	2 (1; 3)	2 (1; 2)	0.672
Dietary calcium (mg/day) ^a^	433 (410; 456)	288 (273; 303)	703 (644; 762)	796 (742; 850)	<0.001	534 (508; 561)	373 (352; 394)	806 (732; 879)	901 (847; 955)	<0.001
Dietary calcium <EAR (%)	89	100	77	59	<0.001	87	97	72	63	<0.001

Notes: ^a^ mean value and 95% confidence interval; ^b^ physical activity categories: low: <600; metabolic energy task (MET)-minutes/week, moderate: 600–1499 MET-minutes/week, moderate-higher: 1500–2999 MET-minutes/week, and high: ≥3000 MET-minutes/week; ^c^ calculated for 399 of mothers because of missing data; EAR: estimated average requirement; *p-*value: level of significance verified by chi^2^ test (categorical variables) or Kruskal–Wallis test (continuous variables of dietary data) or analysis of variance (ANOVA) (continuous variables of body weight and composition data); ns: statistically insignificant; NA: not applicable.

**Table 2:** Distributions (%) of body weight and composition characteristics by dairy-related dietary patterns.

Characteristics	Mothers					Daughters				
	Total	Dairy-Related Dietary Patterns			*p*-Value	Total	Dairy-Related Dietary Patterns			*p*-Value
		Common	Cheese and Fruit Yoghurt	Natural Milk Beverages and Cottage Cheese			Common	Yoghurt and Cheese	Milk and Cheese	
	(*n* = 691)	(*n* = 477)	(*n* = 88)	(*n* = 126)		(*n* = 691)	(*n* = 463)	(*n* = 94)	(*n* = 134)	
*z*-WC (SDs)										
<−1	14	11	23	19		10	10	13	10	
−1–1	70	69	68	72	0.006	76	77	73	74	ns
>1	16	19	9	9		14	13	14	16	
WC ^a^ > 80 cm	52	58	41	39	<0.001	8	7	9	10	ns
*z*-Body fat (SDs)										
<−1	16	13	30	17		15	16	10	15	
−1–1	68	70	64	64	<0.001	69	70	73	66	ns
>1	16	18	7	18		16	14	17	19	
Body fat (%) ^b^										
<14	0	0	0	0		NA	NA	NA	NA	
14–28	21	18	34	23	0.001	NA	NA	NA	NA	
29–32	24	24	31	18		NA	NA	NA	NA	
>32	55	57	35	59		NA	NA	NA	NA	
*z*-WHtR (SDs)										
<−1	15	12	24	18		12	11	14	16	
−1–1	70	71	68	71	0.004	74	77	68	69	ns
>1	15	17	8	10		14	12	18	15	
WHtR > 0.5	45	51	32	35	<0.001	5	5	5	7	
*z*-BMI (SDs)										
<−1	13	11	18	17		12	11	9	17	
−1–1	73	73	75	72	ns	75	77	73	69	ns
>1	14	16	7	11		14	13	18	14	
BMI (kg/m^2^) ^c,d^										
<18.5	1	1	1	2		16	15	12	21	
18.5–24.9	48	43	58	58	0.010	78	78	81	73	
25–29.9	38	41	34	30		6	6	6	5	ns
≥30	13	15	7	10		1	1	1	1	

Notes: *z*-WC: waist circumference *z*-score; SDs: standard deviations; WC: waist circumference; ^a^ waist circumference categorized in accordance with Alberti et al. [41]; *z*-Body fat: body fat *z*-score; ^b^ Body fat categorized by Taton recommendations [43]; WHtR: waist-to-height ratio; *z*-WHtR: waist-to-height ratio *z*-score; ^c^ WHtR categorized by Ashwell et al. recommendations [40]; BMI: body mass index; *z*-BMI: body mass index *z*-score; ^d^ BMI categorized in accordance with International Obesity Task Force (IOTF) standards [42], for female <18 years old according to age-sex-specific BMI cut-offs; *p-*value level of significance verified by chi^2^ test; ns: statistically insignificant; ns: statistically insignificant; NA: not applicable.

**Table 3:** Means (with 95% confidence interval) of body weight and composition characteristics by dairy-related dietary patterns.

Characteristics	Mothers						Daughters					
	Total	Dairy-Related Dietary Patterns			Difference ^a^ between DDPs		Total	Dairy-Related Dietary Patterns			Difference ^a^ between DDPs	
		Common	Cheese and Fruit Yoghurt	Natural Milk Beverages and Cottage Cheese	Cheese and Fruit Yoghurt	Natural Milk Beverages and Cottage Cheese		Common	Yoghurt and Cheese	Milk and Cheese	Yoghurt and Cheese	Milk and Cheese
	(*n* = 691)	(*n* = 477)	(*n* = 88)	(*n* = 126)			(*n* = 691)	(*n* = 463)	(*n* = 94)	(*n* = 134)		
WC (cm)	82.5 (81.7; 83.4)	83.9 (82.9; 84.9)	78.8 (76.7; 80.9)	79.9 (78.2; 81.6)	−5.1 ***	−4.0 ***	69.6 (69.1; 70.1)	69.4 (69.7; 69.1)	69.6 (68.1; 71.0)	69.5 (68.2; 70.8)	0.2	0.1
*z*-WC (SDs)	0.00 (−0.07; 0.07)	0.13 (0.04; 0.23)	−0.34 (-0.54; -0.15)	−0.24 (−0.40; −0.08)	−0.47 ***	−0.37 ***	0.00 (−0.07; 0.07)	0.01 (−0.08; 0.10)	−0.01 (−0.22; 0.21)	−0.02 (−0.21; 0.17)	−0.02	−0.03
Body fat (%)	32.6 (32.3; 33.0)	33.1 (32.6; 33.5)	30.4 (29.4; 31.4)	32.5 (31.6; 33.3)	−2.7 ***	−0.6	23.6 (23.3; 23.9)	23.4 (23.0; 23.8)	24.1 (23.3; 24.9)	23.8 (23.0; 24.6)	0.7	0.4
*z*-Body fat (SDs)	0.00 (−0.07; 0.07)	0.09 (0.01; 0.18)	−0.46 (-0.66; −0.25)	−0.03 (−0.21; 0.15)	−0.55 ***	−0.12	0.00 (−0.07; 0.07)	-0.04 (−0.13; 0.05)	0.13 (−0.06; 0.31)	0.06 (−0.13; 0.25)	0.17	0.10
WHtR	0.50 (0.49; 0.51)	0.50 (0.49; 0.51)	0.48 (0.47; 0.50)	0.51 (0.50; 0.52)	−0.02 ***	0.01 ***	0.42 (0.42; 0.43)	0.42 (0.42; 0.43)	0.42 (0.42; 0.43)	0.43 (0.42; 0.43)	0.00	0.01
*z*-WHtR (SDs)	0.00 (−0.07; 0.07)	0.13 (0.04; 0.22)	−0.35 (−0.54; −0.16)	−0.24 (−0.40; −0.08)	−0.48 ***	−0.37 ***	0.00 (−0.07; 0.07)	−0.01 (−0.10; 0.07)	0.04 (−0.19; 0.26)	0.02 (−0.17; 0.21)	0.05	0.03
BMI (kg/m^2^)	25.7 (25.4; 26.0)	26.1 (25.8; 26.5)	24.3 (23.6; 25.1)	24.8 (24.2; 25.5)	−1.8 ***	−1.3 ***	20.7 (20.5; 20.9)	20.7 (20.5; 20.9)	21.0 (20.5; 21.6)	20.5 (20.0; 21.0)	0.3	−0.2
*z*-BMI (SDs)	0.00 (−0.07; 0.07)	0.11 (0.02; 0.21)	−0.32 (−0.50; −0.15)	−0.20 (−0.36; −0.04)	−0.43 ***	−0.31 ***	0.00 (−0.07; 0.07)	0.00 (−0.09; 0.09)	0.12 (−0.09; 0.32)	−0.08 (−0.27; 0.11)	0.12	−0.08

Notes: WC: waist circumference; *z*-WC: waist circumference *z*-score; SDs: standard deviations; *z*-Body fat: body fat *z*-score; WHtR: waist-to-height ratio; *z*-WHtR: waist-to-height ratio *z*-score; BMI: body mass index; *z*-BMI: body mass index *z*-score; ^a^ Difference was calculated for characteristics’ means as follows: Difference = DDPs‒“Common” pattern; Statistically significant: *** *p* < 0.001.

**Table 4:** Adjusted ^a^ odds ratio (95% confidence interval) of the prevalence of abnormal body weight and composition according to dairy-related dietary patterns and calcium intake.

Characteristics	Mothers				Daughters			
	Dairy-Related Dietary Patterns			Dietary Calcium per 100 mg/Day	Dairy-Related Dietary Patterns			Dietary Calcium per 100 mg/Day
	Common	Cheese and Fruit Yoghurt	Natural Milk Beverages and Cottage Cheese		Common	Yoghurt and Cheese	Milk and Cheese	
	(*n* = 477)	(*n* = 88)	(*n* = 126)	(*n* = 691)	(*n* = 463)	(*n* = 94)	(*n* = 134)	(*n* = 691)
*z*-WC (SDs)								
<−1	1.00	1.85 (0.82; 4.20)	1.87 (0.84; 4.15)	1.01 (0.93; 1.09)	1.00	1.14 (0.55; 2.40)	1.00 (0.65; 1.53)	1.05 (0.99; 1.10)
−1–1	1.00	ref.	ref.	ref.	1.00	ref.	ref.	ref.
>1	1.00	0.40 (0.14; 1.15)	0.46 (0.17; 1.25)	0.90 * (0.83; 0.98)	1.00	1.11 (0.57; 2.18)	1.14 (0.64; 2.04)	NA
WC ^a^ (cm)								
≤80	1.00	ref.	ref.	ref.	1.00	ref.	ref.	ref.
>80	1.00	0.47 * (0.24; 0.92)	0.29 *** (0.14; 0.58)	0.93 * (0.88; 0.99)	1.00	1.19 (0.52; 2.76)	1.29 (0.63; 2.63)	0.96 (0.90; 1.02)
*z*-Body fat (SDs)								
<−1	1.00	1.88 (0.93; 3.84)	2.39 * (1.13; 5.04)	1.04 (0.96; 1.11)	1.00	0.52 (0.24; 1.14)	1.06 (0.59; 1.91)	1.01 (0.96; 1.06)
−1–1	1.00	ref.	ref.	ref.	1.00	ref.	ref.	ref.
> 1	1.00	0.21 (0.04; 1.07)	1.07 (0.41; 2.76)	0.96 (0.87; 1.05)	1.00	1.10 (0.58; 2.09)	NA	1.02 (0.98; 1.07)
Body fat (%) ^b^								
<14	1.00	NA	NA	NA	NA	NA	NA	NA
14–28	1.00	ref.	ref.	ref.	NA	NA	NA	NA
29–32	1.00	0.80 (0.35; 1.85)	0.55 (0.22; 1.34)	0.99 (0.91; 1.09)	NA	NA	NA	NA
>32	1.00	0.40 * (0.16; 0.98)	0.91 (0.37; 2.24)	1.02 (0.94; 1.12)	NA	NA	NA	NA
*z*-WHtR (SDs)								
<−1	1.00	2.18 (0.95; 5.00)	1.83 (0.77; 4.36)	1.01 (0.93; 1.10)	1.00	1.18 (0.57; 2.42)	1.87 * (1.02; 3.43)	1.07 ** (1.02; 1.13)
−1–1	1.00	ref.	ref.	ref.	1.00	ref.	ref.	ref.
>1	1.00	0.36 (0.12; 1.13)	0.59 (0.23; 1.51)	0.91 * (0.84; 0.99)	1.00	1.73 (0.92; 3.26)	1.31 (0.72; 2.39)	1.03 (0.98; 1.08)
WHtR								
≤0.5	1.00	ref.	ref.	ref.	1.00	ref.	ref.	ref.
>0.5	1.00	0.40 ** (0.20; 0.81)	0.52 (0.26; 1.03)	0.95 (0.89; 1.01)	1.00	1.06 (0.38; 2.97)	1.17 (0.51; 2.72)	0.96 (0.89; 1.04)
*z*-BMI (SDs)								
<−1	1.00	2.20 (0.93; 5.22)	1.32 (0.53; 3.27)	1.00 (0.91; 1.09)	1.00	0.87 (0.38; 1.99)	1.67 (0.93; 2.99)	1.06 * (1.00; 1.11)
−1–1	1.00	ref.	ref.	ref.	1.00	ref.	ref.	ref.
> 1	1.00	0.36 (0.09; 1.38)	0.69 (0.26; 1.81)	0.91 * (0.82; 1.00)	1.00	1.58 (0.84; 2.96)	1.30 (0.72; 2.35)	1.02 (0.97; 1.07)
BMI (kg/m^2^) ^c^								
<18.5	1.00	NA	NA	NA	1.00	0.82 (0.40; 1.66)	1.47 (0.87; 2.49)	1.05 * (1.01; 1.10)
18.5 to 24.9	1.00	ref.	ref.	ref.	1.00	ref.	ref.	ref.
25 to 29.9	1.00	0.56 (0.28; 1.13)	0.44 * (0.22; 0.88)	0.92 * (0.87; 0.99)	1.00	1.28 (0.48; 3.38)	1.15 (0.46; 2.86)	0.98 (0.91; 1.06)
≥30	1.00	0.32 (0.08; 1.24)	0.67 (0.22; 2.04)	0.89 * (0.79; 0.99)	1.00	NA	NA	1.00 (0.83; 1.21)

Notes: ^a^ Odds ratio were adjusted for age (continuous variable), education (categorical variable with three categories), place of living (categorical variable with two categories), economic situation (categorical variable with four categories), chronic disease (yes or no), menstrual cycle (yes or no), daily consumption of dairy products during pre-school period (yes or no), daily consumption of dairy products during school period (yes or no), dieting (yes or no), taking calcium supplement (yes or no), physical activity (categorical variable with four categories after completing the missing data in mothers by moda category); *z*-WC: waist circumference *z*-score; SDs: standard deviations; ref.: reference; WC: waist circumference; ^a^ waist circumference categorized in accordance with Alberti et al. [41]; *z*-Body fat: body fat *z*-score; ^b^ Body fat categorized by Taton recommendations [43]; *z*-WHtR: waist-to-height ratio *z*-score; ^c^ WHtR categorized by Ashwell et al. recommendations [40]; *z*-BMI: body mass index *z*-score; ^d^ BMI categorized in accordance with International Obesity Task Force (IOTF) standards [42], for female <18 years old according to age-sex-specific BMI cut-offs; Statistically significant: * *p* < 0.05, ** *p* < 0.01, *** *p* < 0.001; NA: not applicable.

## Supplementary Materials

The following are available online at www.mdpi.com/2072-6643/10/1/90/s1, Table S1: Crude odds ratio (95% confidence interval) of prevalence abnormal body weight and composition according to dairy-related dietary patterns and calcium intake, Table S2: Correlation coefficients (r) between mothers’ and daughters’ body weight and composition characteristics.

## Abbreviations

The following abbreviations are used in this manuscript

ADOS-Ca	a semi-quantitative food frequency questionnaire
ANOVA	analysis of variance
BMI	body mass index
DDPs	dairy-related dietary patterns
DPs	dietary patterns
EAR	estimated average requirement
MET	metabolic energy task
NA	not applicable
ns	statistically insignificant
ORs	odds ratios
ref.	reference
SDs	standard deviations
WC	waist circumference
WHtR	waist-to-height ratio
*z*-BMI	*z*-score of body mass index
*z*-Body fat	*z*-score of body fat
*z*-WC	*z*-score of waist circumference
*z*-WHtR	*z*-score of waist-to-height ratio
