# Supplementary material for: Dairy-Related Dietary Patterns, Dietary Calcium, Body Weight and Composition: A Study of Obesity in Polish Mothers and Daughters, the MODAF Project
Source: Nutrients. 2018 Jan 16;10(1):90. doi: 10.3390/nu10010090 (PMC5793318; doi:10.3390/nu10010090)
Supplement: Supplementary file 1 [file nutrients-10-00090-s001.pdf]

nutrients

Dairy-related Dietary Patterns, Dietary Calcium, Body Weight And Composition: A Study of Obesity in Polish Mothers and Daughters, the MODAF Project

Lidia Wadolowska, Natalia Ulewicz, Kamila Sobas, Justyna W. Wuenstel, Malgorzata A. Slowinska, Ewa Niedzwiedzka, Magdalena Czlapka-Matyasik

Supplementary Materials

Table S1. Crude odds ratio (95% confidence interval) of prevalence abnormal body weight and composition according to dairy-related dietary patterns and calcium intake.

Characteristics	Mothers				Daughters			
	Dairy-related dietary patterns			Dietary calcium per 100 mg/day	Dairy-related dietary patterns			Dietary calcium per 100 mg/day
	Common (n=477)	Cheese and fruit yoghurt (n=88)	Natural milk beverages and cottage cheese (n=126)	(n=691)	Common (n=463)	Yoghurt and cheese (n=94)	Milk and cheese (n=134)	(n=691)
z-WC (SDs)								
<-1	1.00	2.04* (1.14; 3.66)	1.62 (0.95; 2.76)	1.04 (0.99; 1.09)	1.00	1.34 (0.67; 2.69)	1.09 (0.58; 2.06)	1.06* (1.01; 1.11)
-1 to 1	1.00	ref.	ref.	ref.	1.00	ref.	ref.	ref.
> 1	1.00	0.48 (0.22; 1.04)	0.43** (0.22; 0.85)	0.92** (0.86; 0.97)	1.00	1.08 (0.57; 2.06)	1.21 (0.71; 2.09)	1.01 (0.96; 1.06)
WC (cm)								
≤80	1.00	ref.	ref.	ref.	1.00	ref.	ref.	ref.
>80	1.00	0.50** (0.31; 0.79)	0.46*** (0.31; 0.69)	0.93*** (0.90; 0.97)	1.00	1.17 (0.53; 2.63)	1.36 (0.69; 2.65)	0.98 (0.92; 1.04)
z-Body fat (SDs)								
<-1	1.00	2.57*** (1.49; 4.42)	1.50 (0.87; 2.60)	1.02 (0.98; 1.07)	1.00	0.56 (0.27; 1.17)	0.98 (0.53; 1.78)	1.00 (0.96; 1.05)
-1 to 1	1.00	ref.	ref.	ref.	1.00	ref.	ref.	ref.
> 1	1.00	0.42 (0.17; 1.00)	1.11 (0.66; 1.87)	0.96 (0.91; 1.01)	1.00	1.13 (0.62; 2.08)	1.44 (0.86; 2.41)	1.03 (0.99; 1.08)

Table S1. *Cont.*

Body fat (%)[a]								
<14	1.00	2.93 (0.17; 49.78)	NA	1.02 (0.71; 1.45)	NA	NA	NA	NA
14 to 28	1.00	ref.	ref.	ref.	NA	NA	NA	NA
29 to 32	1.00	0.69 (0.38; 1.25)	0.61 (0.33; 1.12)	0.97 (0.92; 1.03)	NA	NA	NA	NA
>32	1.00	0.33*** (0.19; 0.58)	0.82 (0.50; 1.34)	0.98 (0.94; 1.03)	NA	NA	NA	NA
z-WHtR (SDs)								
<-1	1.00	2.07** (1.17; 3.67)	1.51 (0.88; 2.59)	1.03 (0.98; 1.08)	1.00	1.48 (0.76; 2.89)	1.74* (1.00; 3.03)	1.08*** (1.03; 1.13)
-1 to 1	1.00	ref.	ref.	ref.	1.00	ref.	ref.	ref.
> 1	1.00	0.47 (0.21; 1.08)	0.58 (0.31; 1.10)	0.93* (0.88; 0.99)	1.00	1.66 (0.91; 3.05)	1.36 (0.78; 2.38)	1.03 (0.98; 1.07)
WHtR								
≤0.5	1.00	ref.	ref.	ref.	1.00	ref.	ref.	ref.
>0.5	1.00	0.46*** (0.28; 0.74)	0.53** (0.35; 0.79)	0.94*** (0.90; 0.97)	1.00	1.03 (0.31; 3.38)	1.32 (0.60; 2.91)	1.00 (0.92; 1.07)
z-BMI (SDs)								
<-1	1.00	1.56 (0.84; 2.90)	1.49 (0.85; 2.59)	1.02 (0.96; 1.10)	1.00	0.82 (0.37; 1.81)	1.78 (1.03; 3.06)	1.05* (1.00; 1.10)
-1 to 1	1.00	ref.	ref.	ref.	1.00	ref.	ref.	ref.
> 1	1.00	0.42 (0.18; 1.01)	0.71 (0.39; 1.32)	0.91* (0.84; 0.99)	1.00	1.51 (0.83; 2.75)	1.26 (0.72; 2.23)	1.02 (0.98; 1.07)
BMI (kg/m^2)[b]								
<18.5	1.00	1.01 (0.11; 9.21)	1.42 (0.25; 7.96)	0.97 (0.80; 1.17)	1.00	0.74 (0.37; 1.47)	1.46 (0.89; 2.39)	1.04* (1.00; 1.09)
18.5 to 24.9	1.00	ref.	ref.	ref.	1.00	ref.	ref.	ref.
25 to 29.9	1.00	0.63 (0.38; 1.03)	0.56** (0.36; 0.86)	0.95** (0.91; 0.99)	1.00	1.10 (0.44; 2.78)	1.00 (0.42; 2.37)	0.99 (0.92; 1.06)
≥30	1.00	0.34** (0.14; 0.81)	0.51* (0.27; 0.98)	0.91* (0.85; 0.97)	1.00	1.59 (0.16; 15.61)	1.23 (0.13; 12.05)	1.03 (0.86; 1.22)

Notes: z-WC: waist circumference z-score; SDs: standard deviations; z-Body fat: body fat z-score; [a]Body fat categorized by Taton recommendations [43]; z-WHtR: waist-to-height ratio z-score; z-BMI: body mass index z-score; [b]BMI categorized in accordance with International Obesity Task Force (IOTF) standards [42], for female <18 years old according to age-sex-specific BMI cut-offs; Statistically significant: * $p<0.05$, ** $p<0.01$, *** $p<0.001$; NA: not applicable.

Table S2. Correlation coefficients (r) between mothers' and daughters' body weight and composition characteristics.

**Characteristics**	**Pearson's r**	***p*-value**
z-WC (SDs)	0.26	<0.001
z-Body fat (SDs)	0.26	<0.001
z-WHtR (SDs)	0.21	<0.001
z-BMI (SDs)	0.21	<0.001

Notes: z-WC: waist circumference z-score; SDs: standard deviations; z-Body fat: body fat z-score; z-WHtR: waist-to-height ratio z-score; z-BMI: body mass index z-score.
